# An important step to avoid complications with leadless pacemaker implants and retrievals

**DOI:** 10.1016/j.hrcr.2024.07.026

**Published:** 2024-08-15

**Authors:** James E. Ip

**Affiliations:** Department of Medicine, Division of Cardiology, Weill Cornell Medicine, New York Presbyterian Hospital, New York, New York

Nomura and colleagues^1^ have written an interesting case report that highlights one of the potential complications that can occur with leadless pacemaker (LP) implantation using the Abbott Aveir system. They describe a case of recapturing a helix-fixation LP with its delivery catheter during a repositioning attempt. Although the protective sleeve did not advance beyond the middle of the device after redocking the device from tether mode, they proceeded to rotate the handle and device counterclockwise. This resulted in the system becoming stuck because of entrapped tissue that became inadvertently “reeled in.” Fortunately, they were able to release the device and catheter, retrieve the free-floating LP with an Aveir retrieval catheter, and implant a new LP successfully.

The authors should be commended for this illustrative case and their ex vivo figures and videos that demonstrate the importance of avoiding entrapment of tissue between the docking button and the docking cap. This phenomenon can occur with either the delivery catheter (in the case of repositioning the LP before release) as described or the dedicated retrieval catheter (in the case of acute or chronic retrieval).

A sign of this potential scenario is the inability to advance the protective sleeve around the mating point of the LP and the docking cap. The sleeve should be advanced to at least half the length of the LP body without any billowing or resistance (as was shown in Supplemental Video 2). If this is observed, then undocking and redocking of the LP (followed by reevaluation by advancing of the protective sleeve over the device) should be done *before* any counterclockwise rotation of the device.

Another method to ensure that tissue is not inadvertently captured is to advance the protective sleeve over the LP while it is in tether mode with the delivery catheter (or after snaring the LP with the retrieval catheter) and docking the device within the protective sleeve. This technique ensures that tissue is not trapped during the mating process, and it helps to maintain proper coaxial alignment to ensure that the docking button is well seated within the docking cap. Other essential techniques for LP retrieval have been published previously.^2^ Just like when riding trains, it is important to stay clear of the closing doors.
